# Semaphorin 3G exacerbates joint inflammation through the accumulation and proliferation of macrophages in the synovium

**DOI:** 10.1186/s13075-022-02817-7

**Published:** 2022-06-04

**Authors:** Jumpei Shoda, Shigeru Tanaka, Keishi Etori, Koto Hattori, Tadamichi Kasuya, Kei Ikeda, Yuko Maezawa, Akira Suto, Kotaro Suzuki, Junichi Nakamura, Yoshiro Maezawa, Minoru Takemoto, Christer Betsholtz, Koutaro Yokote, Seiji Ohtori, Hiroshi Nakajima

**Affiliations:** 1 Department of Allergy and Clinical Immunology, Graduate School of Medicine, Chiba University, Chiba, Japan; 2 Department of Orthopedic Surgery, Graduate School of Medicine, Chiba University, Chiba, Japan; 3 Department of Endocrinology, Hematology, and Gerontology, Graduate School of Medicine, Chiba University, Chiba, Japan; 4 Department of Medicine, Division of Diabetes, Metabolism and Endocrinology, International University of Health and Welfare, Narita, Japan; 5 Department of Immunology, Genetics and Pathology (IGP), Uppsala University, Uppsala, Sweden

**Keywords:** Rheumatoid arthritis, Semaphorin, Macrophage, Neuropilin-2, Methotrexate

## Abstract

**Objectives:**

Methotrexate (MTX) is an anchor drug for the treatment of rheumatoid arthritis (RA). However, the precise mechanisms by which MTX stalls RA progression and alleviates the ensuing disease effects remain unknown. The aim of the present study was to identify novel therapeutic target molecules, the expression patterns of which are affected by MTX in patients with RA.

**Methods:**

CD4^+^ T cells from 28 treatment-naïve patients with RA before and 3 months after the initiation of MTX treatment were subjected to DNA microarray analyses. The expression levels of semaphorin 3G, a differentially expressed gene, and its receptor, neuropilin-2, were evaluated in the RA synovium and collagen-induced arthritis synovium. Collagen-induced arthritis and collagen antibody-induced arthritis were induced in semaphorin3G-deficient mice and control mice, and the clinical score, histological score, and serum cytokines were assessed. The migration and proliferation of semaphorin 3G-stimulated bone marrow-derived macrophages were analyzed in vitro. The effect of local semaphorin 3G administration on the clinical score and number of infiltrating macrophages during collagen antibody-induced arthritis was evaluated.

**Results:**

Semaphorin 3G expression in CD4^+^ T cells was downregulated by MTX treatment in RA patients. It was determined that semaphorin 3G is expressed in RA but not in the osteoarthritis synovium; its receptor neuropilin-2 is primarily expressed on activated macrophages. Semaphorin3G deficiency ameliorated collagen-induced arthritis and collagen antibody-induced arthritis. Semaphorin 3G stimulation enhanced the migration and proliferation of bone marrow-derived macrophages. Local administration of semaphorin 3G deteriorated collagen antibody-induced arthritis and increased the number of infiltrating macrophages.

**Conclusions:**

Upregulation of semaphorin 3G in the RA synovium is a novel mechanism that exacerbates joint inflammation, leading to further deterioration, through macrophage accumulation.

## Introduction

Rheumatoid arthritis (RA) is an autoimmune disease characterized by chronic destructive synovial inflammation. Recent advances in RA treatment have improved overall therapeutic outcomes. However, a substantial number of patients do not achieve remission even when biologic disease-modifying anti-rheumatic drugs (biologic DMARDs, bDMARDs) or target synthetic DMARDs (tsDMARDs) accompanied by methotrexate (MTX) are used [1]. Additionally, these drugs cause a heightened risk of serious infections [2, 3]. This is partly because bDMARDs, tsDMARDs, and MTX inhibit the activation of a wide variety of immune cells. In this regard, it is crucial to select target pathways to reduce the severity and frequency of adverse events while guaranteeing the efficacy of RA treatment strategies.

MTX is an anchor drug for RA treatment [4, 5]. MTX is a folate derivative that inhibits nucleotide synthesis, resulting in decreased immune cell proliferation [6]. Furthermore, several studies revealed other mechanisms by which MTX suppresses immune responses [7–10]. For example, MTX inhibits 5-aminoimidazole-4-carboxamide ribonucleotide (AICAR) transferase (ATIC), causing the accumulation of intracellular AICAR. AICAR accumulation promotes adenosine release and diminishes inflammation [10]. However, further understanding of the pharmacological action of MTX is required. Therefore, we aimed to analyze the molecular mechanisms improving RA pathology and identify novel therapeutic targets.

Herein, we examined the gene-expression profiles of peripheral blood CD4^+^ T cells in 28 treatment-naïve RA patients before and after MTX treatment and evaluated the roles of one of the differentially expressed genes, semaphorin 3G (Sema3G), in murine experimental arthritis models.

## Methods

### Patients

Twenty-eight patients who fulfilled the American College of Rheumatology 1987 revised criteria for the classification of RA and who had never received any DMARDs were recruited for DNA microarray analysis. MTX treatment was initiated for these patients as standard clinical care. Patient characteristics are listed in Table 1. For Sema3G detection, eight RA and eight osteoarthritis (OA) patients who underwent joint surgery were recruited, and the synovium was trimmed from the surgical specimen. The procedure was approved by the Ethics Committee of Chiba University (reference numbers 872 and 3880) and written informed consent was obtained in accordance with the Declaration of Helsinki.

**Table 1:** Patient characteristics

Variables	Treatment naïve RA (*n* = 28)
Age, median (IQR) years	59.5 (47.5–67)
Female, *n* (%)	20 (71.4)
Disease duration, median (IQR) months	7 (3–24)
RF positive, *n* (%)	25 (89.2)
ACPA positive, *n* (%)	26 (92.9)
Maximum dose of MTX, median (IQR) mg/week	10 (8–11)
Dose of prednisolone at baseline, median (IQR) mg/day	0 (0–0)
ESR, median (IQR) mm/h pre-treatment	31 (14–46)
ESR, median (IQR) mm/h post-treatment	11 (9–27)
CRP, median (IQR) mg/dl pre-treatment	0.7 (0.3–1.5)
CRP, median (IQR) mg/dl post-treatment	0.1 (0.1–0.4)
DAS28-ESR, median (IQR) pre-treatment	4.21 (3.83–5.02)
DAS28-ESR, median (IQR) post-treatment	2.54 (2.02–4.39)

*IQR* Inter-quartile range, *RF*, Rheumatoid factor, *ACPA* Anti-cyclic citrullinated protein antibody, *MTX* Methotrexate, *ESR* Erythrocyte sedimentation rate, *CRP* C-reactive protein, *DAS28* Disease Activity Score 28

### Mice

C57BL6/J mice were purchased from Japan CLEA. Sema3G-deficient (Sema3G^-/-^) mice have been described previously [11]. Mice were housed in specific pathogen-free facilities. All animal experiments were conducted in accordance with the Animal Care and Use Committee of Chiba University.

### Collagen-induced arthritis and collagen antibody-induced arthritis

For collagen-induced arthritis (CIA), 8- to 12-week-old mice were immunized intradermally at the base of the tail with 100 μl of chicken type II collagen (10 μg/ml) (Chondrex) emulsified with complete Freund’s adjuvant (20 μg/ml *Mycobacterium butyricum*) (Chondrex). Three weeks later, the mice received the same immunization. After the second immunization, the clinical scores were recorded three times a week. For collagen antibody-induced arthritis (CAIA), 8- to 12-week-old mice received an intravenous injection of 100 μl ArthritoMab (MD Bioproducts). On day 4, mice were intraperitoneally injected with 100 μg LPS. The clinical scores were recorded daily. Clinical symptoms were graded based on the following criteria: 0 = no joint swelling; 1 = mild joint swelling/erythema of the ankle, wrist, or digit; 2 = moderate joint swelling; 3 = severe joint swelling; and 4 = severe joint swelling with ankylosis. The clinical scores were obtained by summing the scores for each limb.

### Antibodies

Anti-Sema3G polyclonal antibodies were purchased from MyBioSource. Anti-neuropilin2 (Nrp2) polyclonal antibodies conjugated with FITC were purchased from Alomone Labs (#ANR-062). BV785- or BV421-conjugated anti-CD45 (104), PerCP-Cy5.5-conjugated anti-CD3ε (145-2C11), APC-conjugated anti-B220 (RA3-6B2), BV421-conjugated anti-CD11b (M1/70), BV711-conjugated anti-F4/80 (BM8), PE-conjugated anti-CD86 (GL-1), PerCP-Cy5.5-conjugated anti-MHC-II (M5/114.15.2), and Zombie Aqua were purchased from BioLegend.

### Recombinant Fc-tagged Sema3G production

The Sema3G expression vector has been described previously [12]. The vector was transfected into 293T cells using Lipofectamine LTX (Thermo Fisher Scientific). Cells were cultured in DMEM supplemented with ultra-low IgG FBS (Gibco). The culture supernatant was collected and dialyzed using Amicon Ultra 100 K (Merck). The dialyzed supernatant was further purified with a Protein G column (Cytiva), and the buffer was exchanged with PBS. Sema3G production was confirmed by Coomassie blue staining and western blotting.

### Immunohistochemistry

Formalin-fixed, paraffin-embedded samples were subjected to Sema3G immunostaining. After dewaxing and rehydration, the samples were boiled in 0.01M citric acid buffer (pH6.0) for 10 min. The samples were then washed and blocked in 3% bovine serum albumin (BSA) solution, followed by an overnight primary antibody reaction (antibody dilution rate = 1:50). After washing, the samples were subjected to secondary antibody staining and visualization with DAB using a liquid DAB substrate/chromogen system (DACO).

### Bone marrow-derived macrophage culture

Bone marrow cells were cultured in the presence of recombinant M-CSF (50 ng/ml) (Pepro Tech) to prepare bone marrow-derived macrophages (BMMs). For Nrp2 expression analysis, BMMs were stimulated with 100 ng/ml LPS (Sigma Aldrich), 10 ng/ml IFN γ (Pepro Tech), or 20 ng/ml IL-4 (Pepro Tech) for 2 days. For RNA-seq analyses, LPS-stimulated BMMs were cultured in the presence or absence of 100 ng/ml recombinant Sema3G for 18 h. For cell-proliferation analysis, 5×10^4^ BMMs were seeded in a 24-well plate and cultured with or without recombinant Sema3G for 48 h. Cells were incubated with EdU for 2 h, followed by Hoechst 33342 and EdU staining (Click-iT EdU assay; Thermo Fisher Scientific).

### Transwell migration assay

Transwell migration assays were performed as previously described [13]. Briefly, the upper chambers with 8 μm pore size membranes were filled with LPS-stimulated BMMs in 1% FBS-supplemented medium. The lower chambers were filled with 700 mL of 1% FBS-supplemented medium with PBS, Sema3G, or MCP1 (BioLegend), as indicated. After 6-h stimulation, cells on the underside of the membrane were stained with Hoechst.

### Flow cytometry

Joint-infiltrating cells were isolated as described previously [14]. Cells were first stained with Zombie Aqua to exclude dead cells from the analyses and subsequently incubated with anti-CD16/32 antibody (BioLegend) to block Fc receptors. The samples were then stained with antibodies against CD45, CD3ε, B220, CD11b, F4/80, and Nrp2 at 4°C for 30 min. For the evaluation of BMMs, adhered cells were detached using 5 mM EDTA in PBS and then stained with anti-Nrp2 antibody. The samples were analyzed on a Canto II or Fortessa X-20 (BD Biosciences).

### Microarray analysis

Peripheral blood samples were prepared from MTX-treated patients, as previously described [15], and CD4^+^ T cells were further enriched using a human CD4^+^ T cell isolation kit (Miltenyi) [16–18]. The purity of the CD4^+^ T cells was > 95%. Total RNA was extracted using Isogen solution (Nippon Gene). DNA microarray analysis was performed using a Quick Amp labeling kit and Whole Human Genome DNA Microarray 4× 44 K, according to the manufacturer's protocol (Agilent). The signal intensity was normalized by adjusting the data to the 75th percentile. The linear models for microarray data (Limma) package of the R project were used to identify candidate probes [19].

### RNA-seq analysis

Total RNA was purified using the TRIzol reagent. RNA-seq libraries were prepared using a QuantSeq 3′ mRNA-Seq Library Prep Kit FWD for Illumina and UMI Second Strand Synthesis Module for QuantSeq FWD (Lexogen). Sequencing was performed on an Illumina NexSeq500 (Illumina) in 75-base single-end mode. The obtained reads were mapped to the mm10 genome and UMI counts were measured using Strand NGS (Agilent). Expression data were normalized, and differentially expressed genes were identified using the EdgeR software.

### ELISA

The serum levels of IL-6 and TNFα were measured using a mouse ELISA MAX Standard IL-6 kit (BioLegend) and a mouse ELISA MAX Standard TNFα kit (BioLegend).

### Statistical analyses

An unpaired, paired *t*-test or one-way analysis of variance (ANOVA) was used for data analysis using Prism9 (GraphPad). Clinical scores were assessed using a two-way ANOVA test. Statistical significance was set at *P* < 0.05.

### Data availability

Microarray and RNA-seq data were deposited in the Gene Expression Omnibus and were accessible through GSE176440 and GSE176438. The data are publicly available for publication.

## Results

### Semaphorin 3G expression found to be upregulated in the inflamed joint

**Fig. 1:**
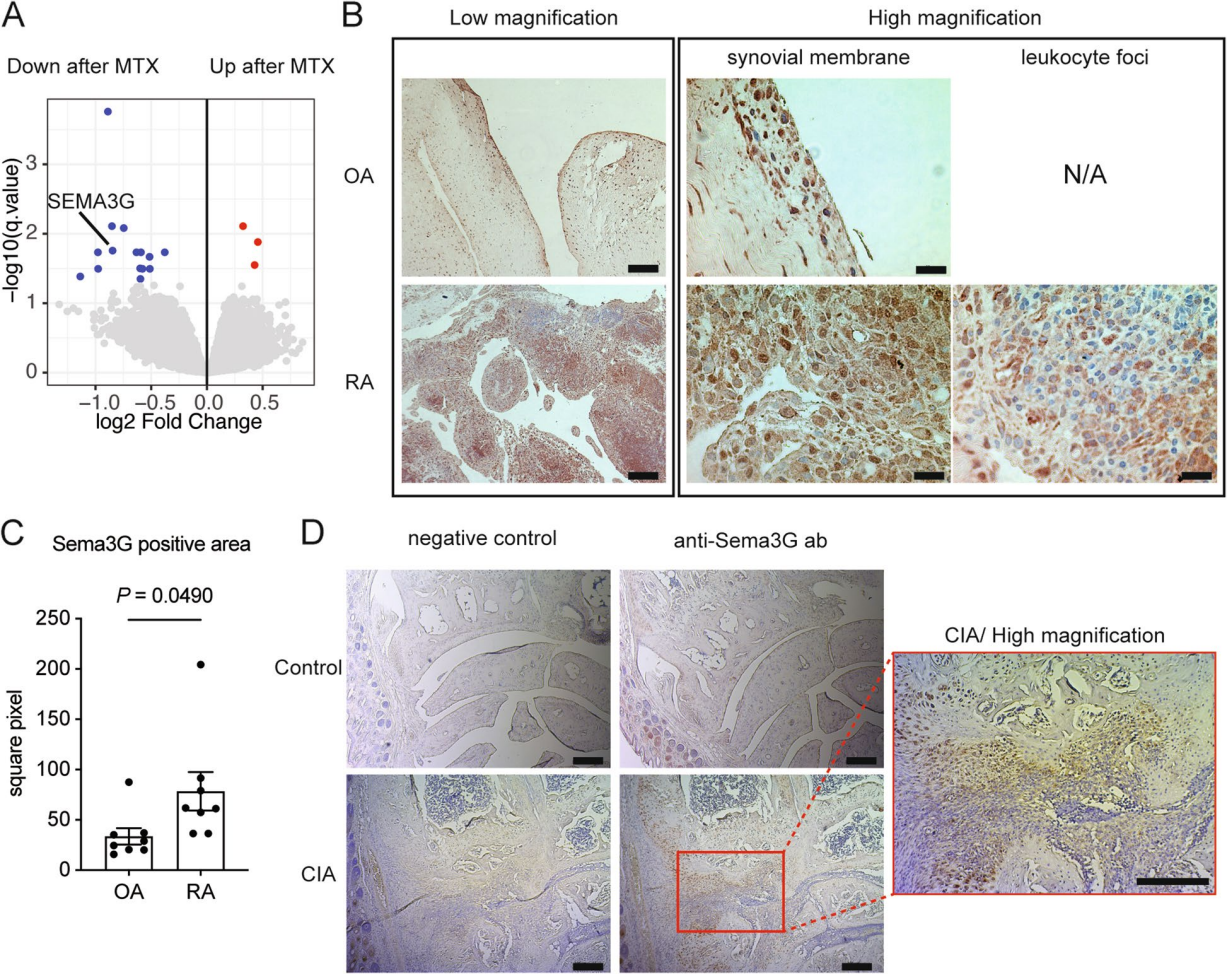
The enhanced expression of Sema3G in the inflamed synovium in humans and mice. **A** Genes differentially expressed before and after MTX treatment. Genes differentially expressed are highlighted in red (upregulated after MTX) or blue (downregulated after MTX). **B** Sema3G expression in the synovium of OA or RA patients. The synovium specimens were stained with anti-Sema3G antibody and visualized with DAB. Bars indicate 200 μm (Low magnification) or 20 μm (high magnification). **C** The cumulative data of the Sema3G-positive area. Data are expressed as the means ± SEM. The statistical analyses were performed using an unpaired *t*-test. **D** Sema3G expression in the synovium of CIA. The hind paws of the control and CIA-induced mice were stained with anti-Sema3G antibody. The representative data are shown. Similar results were obtained in three independent experiments. Bars indicate 200 μm

We first performed DNA microarray analyses of peripheral blood CD4^+^ T cells before and after MTX treatment to identify novel mechanisms by which MTX improves RA disease activity. We identified several differentially expressed genes (DEGs) and focused on Sema3G (Fig. 1A). Sema3G belongs to the class 3 semaphorin family; it has been ascertained that Sema3G plays important roles in neural and vascular development [20, 21]. Although several studies have suggested that semaphorins are pivotally involved in autoimmune diseases [22, 23], the role of Sema3G in this context is yet to be elucidated. Therefore, we analyzed Sema3G expression in human synovium obtained from patients with RA and OA. As described previously, the OA synovium was observed to be a monolayer, and synoviocytes and fibroblast-like spindle-shaped cells were slightly positive for Sema3G (Fig. 1B). In contrast, multilayered synoviocytes were observed in the RA synovium and expressed substantial levels of Sema3G (Fig. 1B). In addition, synovium-infiltrating leukocytes expressed Sema3G (Fig. 1B). We compared the Sema3G-positive area in the synovial tissue and found that the Sema3G-positive area was significantly larger in the RA synovium than in the OA synovium (Fig. 1C).

Next, we assessed Sema3G expression in CIA. Non-arthritic control mice had minimal synovium in the wrist joints with no Sema3G signals (Fig. 1D). CIA-induced mice showed thick synovium with abundant Sema3G signals (Fig. 1D). These results implied that Sema3G is involved in the pathogenesis of joint inflammation.

### Activated macrophages found to express neuropilin-2, a receptor for Sema3G, in the inflamed joint

**Fig. 2:**
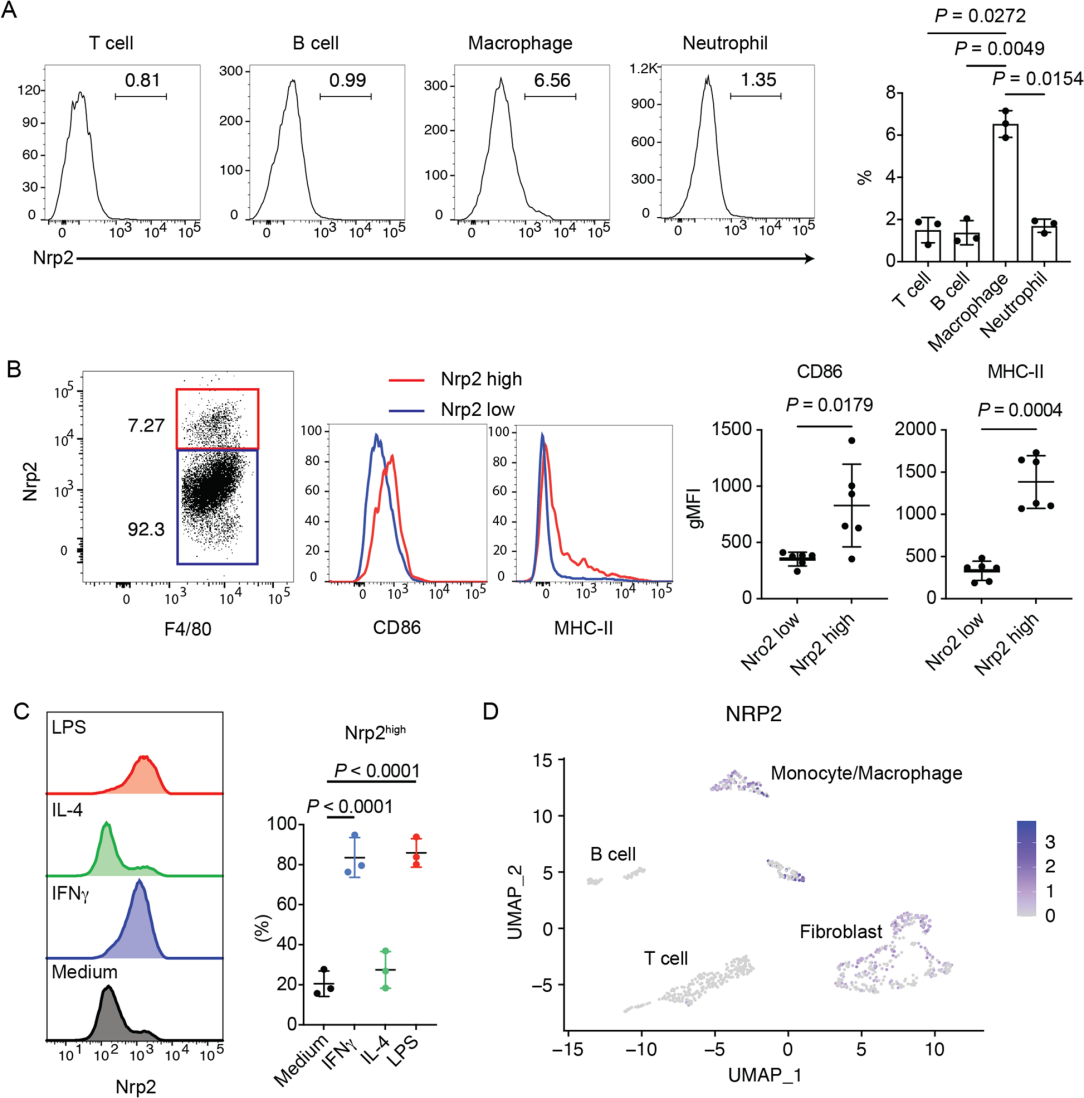
Nrp2 expression in activated macrophages during joint inflammation. **A** Nrp2 expression in immune cells in the CIA synovium. The hind paws of CIA-induced mice were digested and subjected to flow-cytometric analysis. The representative histograms of Nrp2 staining and the cumulative data are shown. Data are expressed as the means ± SD. *N* = 3 from three independent experiments. **B** The characteristics of Nrp2-high macrophages. The representative histograms of CD86 and MHC class II expression on joint-infiltrating macrophages (left) and their cumulative data (right) are shown. *N* = 6 from two independent experiments. **C** Nrp2 expression on BMMs. BMMs were cultured under the indicated conditions, and Nrp2 expression was determined by flow cytometry. The representative histograms and the cumulative data are shown. *N* = 3 from three independent experiments. **D** NRP2 expression in synovium-infiltrating cells in RA. The deposited single-cell RNA-seq data were reanalyzed, and several cell types were defined by UMAP. NRP2 expression is denoted by purple dots. The statistical analyses were performed using an unpaired t-test

Previous studies have shown that Nrp2 and plexin complex [24] functions as receptors for full-length Sema3G. To clarify the cell types that respond to Sema3G in inflamed joints, joint-infiltrating immune cells were collected from CIA-induced mice and analyzed for Nrp2 expression. Among several immune cells, macrophages showed the highest percentage of the Nrp2-high population (Fig. 2A). Notably, Nrp2-high macrophages expressed higher levels of CD86 and MHC class II, which are representative activation markers (Fig. 2B). It was consequently inferred that signals transmitted via Nrp2 might be involved in macrophage activation. Next, BMMs were cultured under various conditions to determine the type of macrophages that preferentially express Nrp2. Without any stimulation, only 20% of the BMMs were found to be Nrp2-high (Fig. 2C). Importantly, IFNγ or LPS stimulation efficiently induced Nrp2 expression but IL-4 did not (Fig. 2C). As IFNγ and LPS have a strong association with type 1 inflammation (i.e., M1 macrophages), this finding aligns with the concept that the Sema3G–Nrp2 axis plays a pathogenic role in RA. We also sought to determine if macrophages in the human RA synovium express Nrp2. To address this issue, we reanalyzed publicly available single-cell RNA-seq data of the RA synovium [25]. Consistent with the data obtained from the CIA model, the CD14-positive monocyte/macrophage population was found to express Nrp2 (Fig. 2D); however, other immune cells, such as T cells and B cells, did not express Nrp2. Together, these results suggested that macrophages respond to Sema3G in the inflamed joints.

### Inflammatory arthritis models were mild in Sema3G^−/−^ mice

**Fig. 3:**
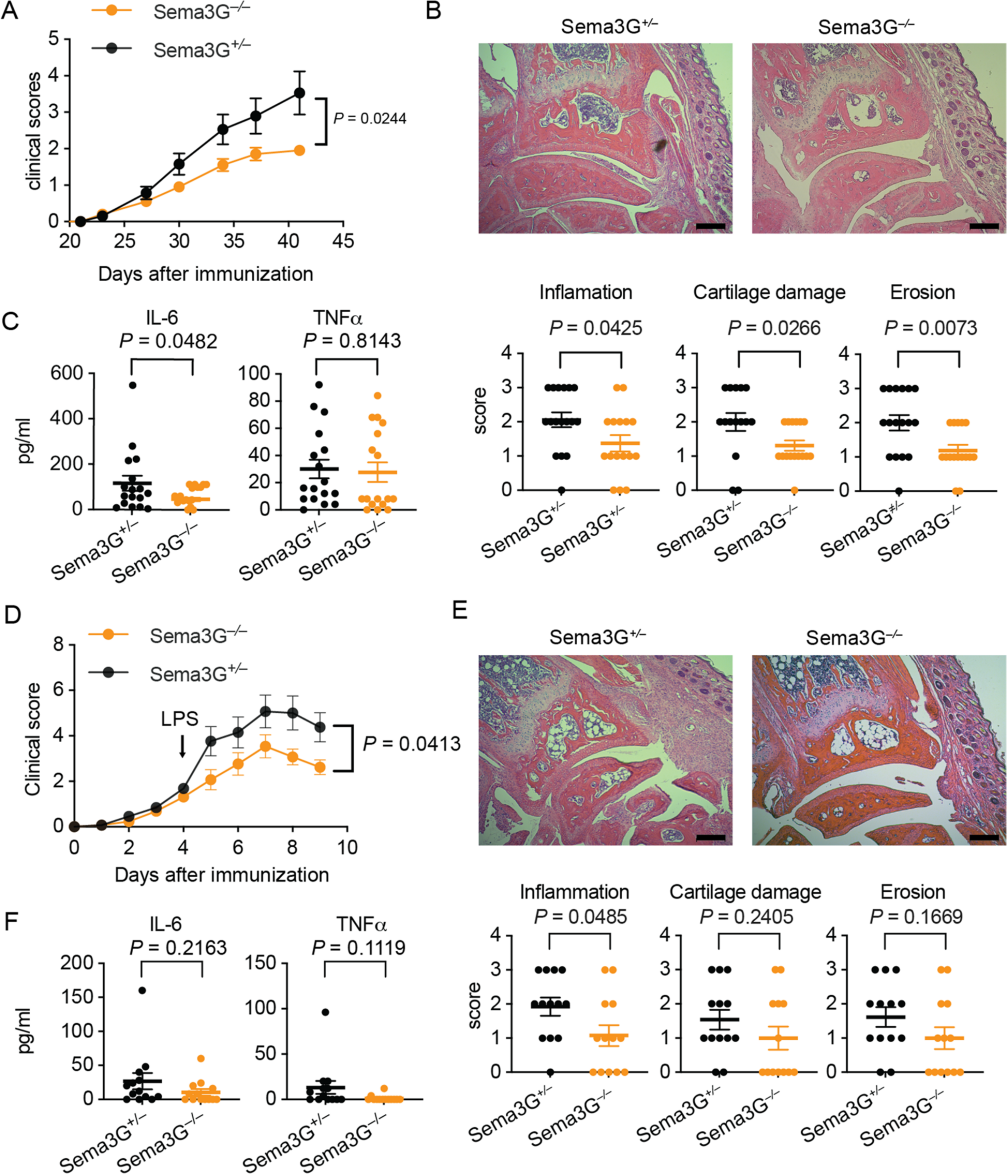
Attenuated joint inflammation in Sema3G-deficient mice. **A** The clinical score of Sema3G-deficient (Sema3G^−/−^) mice and their littermate controls (Sema3G^+/−^) in CIA. Data were obtained from four independent experiments (*N* = 17 in each group). **B** The pathological scores of CIA. The front paws were subjected to H&E staining, and the inflammation, the cartilage damage, and the erosion scores were separately assessed. **C** The inflammatory cytokines in sera. The sera were collected on day 42 and subjected to ELISA to measure IL-6 and TNFα. **D** The clinical score of Sema3G^−/−^ mice and Sema3G^+/−^ in CAIA. Data were obtained from four independent experiments (*N* = 13 in each group). **E** The pathological scores of CAIA. The front paws were subjected to H&E staining, and the inflammation, the cartilage damage, and the erosion scores were separately assessed. **F** The inflammatory cytokines in sera. The sera were collected on day 9 and subjected to ELISA to measure IL-6 and TNFα. Data are expressed as the means ± SEM. For the analyses of the clinical score, 2-way ANOVA was used. For the pathological scores, an unpaired *t*-test was used

To address the role of Sema3G in arthritis, CIA was induced in Sema3G^−/−^ mice and their littermate controls. We found that Sema3G-heterozygous mice (Sema3G^+/−^ mice) expressed a comparable level of *Sema3g* in the spleen, kidney, and pancreas to wild-type mice (data not shown); Sema3G^+/−^ mice were used as controls. Notably, the clinical score was significantly lower in Sema3G^−/−^ mice than in control Sema3G^+/−^ mice (Fig. 3A). Moreover, the pathological scores were lower in Sema3G^−/−^ mice (Fig. 3B). We also measured IL-6 and TNFα in the sera to assess systemic inflammatory conditions in these mice. While there was no difference in TNFα levels, serum IL-6 levels were significantly lower in Sema3G^−/−^ mice (Fig. 3C).

It is well known that both innate and adaptive immune responses are required to develop CIA [26]. Since we next sought to dissect the mechanisms by which Sema3G aggravates CIA, we employed CAIA, in which adaptive immunity is less critical in the development of arthritis [27]. In this model, an anti-collagen antibody was injected intravenously into mice on day 0, and LPS was injected intraperitoneally to boost inflammation on day4. We found that Sema3G^−/−^ mice showed attenuated clinical scores in CAIA when comparing the overall disease course with control Sema3G^+/−^ mice (Fig. 3D). Notably, there was no drastic change in the deterioration rate after LPS stimulation in Sema3G^−/−^ mice, whereas control mice showed obvious responses to LPS stimulation (Fig. 3D). Indeed, there was no statistical difference in the clinical scores until day 4 (*P* = 0.233). These findings suggested that Sema3G^−/−^ mice are hyporesponsive to LPS but normally respond to inflammation-inducing antibodies.

Next, we analyzed the histological scores of CAIA. While there were no statistical differences in cartridge damage and erosion scores, the inflammatory score was significantly lower in Sema3G^−/−^ mice (Fig. 3E). We also observed lower IL-6 and TNFα levels in the sera of Sema3G^−/−^ mice; however, the difference was not statistically significant (Fig. 3F). Taken together, these results indicated that Sema3G exacerbates inflammatory arthritis and acts primarily on innate immunity.

### Sema3G promoted joint inflammation through macrophage migration and proliferation

**Fig. 4:**
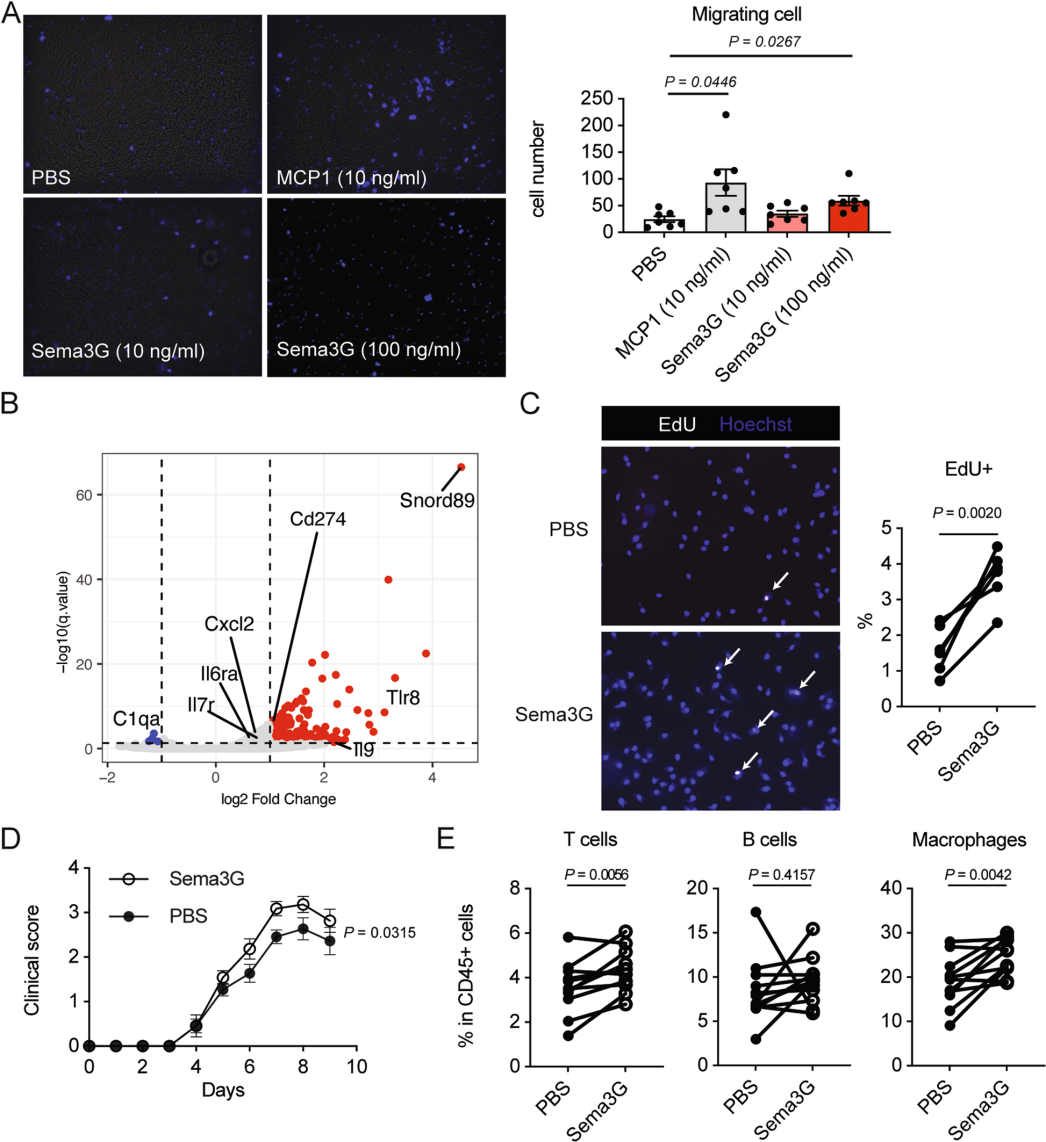
Enhanced macrophage proliferation by Sema3G. **A** Chemotaxis assay. LPS-stimulated BMMs were subjected to a transwell migration assay. PBS (negative control), MCP1 (positive control), or various concentrations of Sema3G were added to the lower chambers. Cell migration towered to the lower chamber was determined by Hoechst staining. The pictures were taken for five different areas, and Hoechst-positive round cell numbers were counted. *N* = 7 from three independent experiments. One-way ANOVA test, followed by Dunnet test, was used for the statical analysis. **B** The volcano plot of Sema3G-stimulated BMMs. Genes differentially expressed are plotted as red dots (upregulated by Sema3G) or blue dots (downregulated by Sema3G). Some immune-related gene names are labeled. **C** EdU uptake in Sema3G-stimulated BMMs. Sema3G-stimulated or control (PBS) BMMs were pulsed with 10 μM EdU for 2 h, and EdU and Hoechst were detected by immunofluorescent analysis. The pictures were taken for ten different areas, and the percentage of EdU-positive cells over Hoechst-positive cells was calculated. Data were obtained from three independent experiments. *N* = 6. **D** The clinical score of Sema3G- or PBS-injected paws during CAIA. WT mice were subjected to CAIA. Sema3G (100 ng) was injected into the right footpad, and PBS was injected into the left footpad daily from day 3 to day 9. *N* = 11 from two independent experiments. **E** Infiltrating cell subsets to each paw on day 9 of CAIA. The hind paws were digested, and cells recovered were analyzed by flow cytometry. The percentages of T cells, B cells, and macrophages are shown. A paired *t*-test was used for the statistical analyses

We sought to determine the mechanism by which Sema3G exacerbates inflammatory arthritis. First, we performed a transwell migration assay on LPS-stimulated BMMs to analyze the chemotactic properties of Sema3G, because semaphorins are best characterized as neuron guidance factors. Interestingly, we found that Sema3G promotes macrophage migration to a certain extent; however, its chemotactic property was milder than that of MCP1 (Fig. 4A).

Next, we performed a transcriptome analysis of Sema3G-stimulated BMMs to further understand the molecular mechanisms by which Sema3G drives joint inflammation. In this experiment, LPS-stimulated BMMs were cultured with or without recombinant Sema3G for 18 h and subjected to RNA-seq analysis. Numerous genes were upregulated by Sema3G stimulation, and among these, *Snord89* was highly expressed in Sema3G-stimulated BMMs (Fig. 4B). Snord89 is a non-coding RNA, and it has been reported that Snord89 is related to cell proliferation through Myc activation [28]. Therefore, we hypothesized that Sema3G upregulates Snord89 to facilitate macrophage proliferation, resulting in aggravation of arthritis. To test this hypothesis, we analyzed macrophage proliferation upon Sema3G stimulation. In this experiment, LPS-stimulated BMMs were cultured in the presence or absence of Sema3G for 48 h. Because BMMs adhered to plastic surfaces very firmly and it was difficult to count the number of cells with a hemocytometer, BMMs were incubated with EdU for the last 2 h, and de novo DNA synthesis was evaluated by measuring the incorporation of EdU. As shown in Fig. 4B, the percentage of EdU-positive cells among Hoechst-positive cells was increased in Sema3G-stimulated BMMs compared to that in PBS-stimulated BMMs (Fig. 4C), indicating that Sema3G promotes cell proliferation. Collectively, the enhanced macrophage proliferation by Sema3G may be a mechanism that accelerates joint inflammation.

Finally, we assessed whether the local administration of Sema3G affects the severity of arthritis and macrophage migration and proliferation in vivo. CAIA was induced in wild-type mice, and 100 ng of recombinant Sema3G (10 μl) was injected daily into the footpad of the left side. As a control, 10 μl of PBS was injected daily into the footpad of the right side. Consistent with the in vitro data, Sema3G-injected footpads showed a higher clinical score than did PBS-injected footpads (Fig. 4D). In addition, the percentage of macrophages was significantly higher in the Sema3G-injected left footpads than in the PBS-injected right footpads (Fig. 4E). The frequency of T cells was also increased in the Sema3G-injected footpads (Fig. 4E). These results indicated that Sema3G promotes macrophage migration and proliferation, both in vivo and in vitro.

## Discussion

In the present study, we elucidated the role of Sema3G in the pathogenesis of inflammatory arthritis. We identified Sema3G as one of the genes downregulated by MTX treatment in RA patients. We also found that Sema3G is expressed in inflamed joints in humans and mice. Activated macrophages express Nrp2, a Sema3G receptor. In vivo, Sema3G^−/−^ mice displayed mild CIA and CAIA, and Sema3G administration exacerbated arthritis in CAIA. Mechanistically, Sema3G promotes macrophage migration and proliferation.

Semaphorins were the first to be identified as neural guidance proteins [29]. Semaphorins are largely classified into eight classes: only class 3 semaphorins are secreted, whereas the rest of the classes are transmembrane proteins in mammals [30]. Recent studies revealed their crucial roles in cardiovascular growth [31], bone homeostasis [32], and immune responses [22, 33]. Sema3G was initially defined as a repulsive factor for sympathetic axons [24] and is now linked to several physiological and pathological processes [20, 34–36]. We previously reported a protective role of Sema3G in LPS-induced kidney injury [36]. In podocytes, Sema3G suppresses the production of inflammatory cytokines and chemokines such as IL-6 and CCL2 upon LPS stimulation. In the present study, we performed an unbiased RNA-seq analysis of Sema3G-stimulated BMMs and found no difference in *IL6* level (data not shown). Therefore, it is plausible that Sema3G exerts cell type-specific functions during inflammation. Further studies are essential to understand how Sema3G influences the immune responses in each cell type.

Macrophages play various roles in inflammatory arthritis pathogenesis. Macrophages are classified into two subsets, namely M1 and M2 macrophages. M1 macrophages are considered pro-inflammatory and have been implicated in the pathogenesis of autoimmune diseases, whereas M2 macrophages have anti-inflammatory potential [37]. Macrophages are one of the most abundant immune cell types that infiltrate the synovium in human RA. Reportedly, RA synovial fluid contains more M1 macrophages than M2 macrophages [38]. Furthermore, numerous studies have revealed that the number of M1 macrophages correlates with disease activity and joint damage [38, 39]. As observed in humans, M1 macrophages have pathogenic roles in murine arthritis models [40–42]. These findings indicated that M1 macrophages are involved in the pathogenesis of RA.

In this regard, we showed that Nrp2 expression in BMMs is significantly elevated following LPS or IFNγ stimulation (Fig. 2C), which favors M1 macrophage differentiation. Furthermore, Nrp2-high macrophages express M1 markers in the synovium (Fig. 2B, C). These activation markers are typically expressed in M1 macrophages, and Nrp2-high macrophages may exhibit an M1 phenotype. In contrast, the M2 macrophage-skewing cytokine IL-4 did not promote Nrp2 expression. These findings indicated that M1-like pro-inflammatory macrophages preferentially respond to Sema3G and proliferate in the inflamed synovium, suggesting that Sema3G is a secreted pro-inflammatory protein in the arthritic joint.

Interestingly, Sema3G^−/−^ mice were resistant to LPS stimulation, although Sema3G^−/−^ mice showed normal initial response to an anti-collagen antibody during CAIA (Fig. 3D). It has been reported that activation of the alternative complement pathway accompanied by IgG-Fc receptor coupling is vital for the induction of arthritis [43]. Anti-collagen antibodies are deposited on the cartridge surface and recognized by FcRγIII-bearing cells, such as neutrophils, mast cells, and macrophages. Activation of the antibody-FcRγIII and complement-C5aR pathways promotes the production of inflammatory cytokines, such as IL-6 and TNFα, resulting in joint inflammation. Interestingly, RNA-seq analysis revealed that Sema3G stimulation did not result in upregulated *FcR*γ*III* or *C5aR* expression in BMMs (data not shown). Therefore, we hypothesized that Sema3G plays a negligible role in antibody-mediated immune responses but directly affects macrophage migration and proliferation. This feature may be beneficial when considering Sema3G as a therapeutic target, because antibody-mediated immune responses are essential for pathogen clearance [44].

The present study has several limitations. First, although we identified that Sema3G expression is downregulated by MTX treatment, we could not address the molecular mechanism underlying the MTX-mediated suppression of Sema3G expression because of the difficulty of in vitro experiments using MTX. Second, Sema3G-mediated immune regulation during arthritis has not been addressed adequately. While we found that Nrp2 is expressed in activated macrophages, some lymphoid cells and neutrophils were also positive for Nrp2 in the synovium. Previous studies have revealed the crucial roles of semaphorin family members in T cell differentiation and activation [45–50]. Indeed, we found that T cells increased in the inflamed joint upon local Sema3G administration (Fig. 4E). Therefore, it is possible that the Sema3G–Nrp2 axis also plays a role in T cell-mediated immune responses during arthritis. Fibroblasts in the human synovium also express NRP2 mRNA (Fig. 2D), although we did not analyze non-immune cells in the present study. Previous studies have suggested that signaling through neuropilins promotes myofibroblast formation [51]. Thus, it is possible that Sema3G also affects the phenotype of fibroblasts in RA pathogenesis. Third, we could not directly assess the therapeutic potential of Sema3G neutralization. Although the severity of CIA and CAIA was mitigated in Sema3G^−/−^ mice, antibody-mediated neutralization of Sema3G after the initiation of arthritis would be valuable in determining whether Sema3G could serve as a therapeutic target. Further studies are warranted to address this issue when a neutralizing antibody against Sema3G is available.

## Conclusions

Sema3G is implicated in the pathogenesis of RA, and the neutralization of Sema3G may favor the development of a novel therapeutic approach belonging to a new category.

## Data Availability

The datasets used and/or analyzed during the current study are available from the corresponding author upon reasonable request.

## Abbreviations

ACPA: Anti-cyclic citrullinated protein antibody
AICAR: 5-Aminoimidazole-4-carboxamide ribonucleotide
BMMs: Bone marrow-derived macrophages
CAIA: Collagen antibody-induced arthritis
CIA: Collagen-induced arthritis
CRP: C-reactive protein
DAS28: Disease Activity Score 28
DEG: Differentially expressed genes
DMARDs: Disease-modifying anti-rheumatic drugs
ESR: Erythrocyte sedimentation rate
MTX: Methotrexate
Nrp2: Neuropilin-2
OA: Osteoarthritis
RA: Rheumatoid arthritis
RF: Rheumatoid factor
Sema3G: Semaphorin3G
tsDMARD: Target synthetic DMARD
